# Protective Effect of *N*-Acetylserotonin against Acute Hepatic Ischemia-Reperfusion Injury in Mice

**DOI:** 10.3390/ijms140917680

**Published:** 2013-08-29

**Authors:** Shuna Yu, Jie Zheng, Zhengchen Jiang, Caixing Shi, Jin Li, Xiaodong Du, Hailiang Wang, Jiying Jiang, Xin Wang

**Affiliations:** 1 Departments of Anatomy, Weifang Medical University, Weifang 261053, China; E-Mails: yushn@wfmc.edu.cn (S.Y.); medicaljzc@163.com (Z.J.); shicaixing123@126.com (C.S.); lijin@wfmc.edu.cn (J.L.); duxd@wfmc.edu.cn (X.D.); wanghailiang0781@sina.com (H.W.); 2 Departments of Pathology, Weifang Medical University, Weifang 261053, China; E-Mail: zhengj@wfmc.edu.cn; 3 Department of Neurosurgery, Brigham and Women’s Hospital, Harvard Medical School, Boston, MA 02115, USA

**Keywords:** *N*-acetylserotonin, newborn mouse, hepatic ischemia-reperfusion injury, apoptosis

## Abstract

The purpose of this study was to investigate the possible protective effect of *N-*acetylserotonin (NAS) against acute hepatic ischemia-reperfusion (I/R) injury in mice. Adult male mice were randomly divided into three groups: sham, I/R, and I/R + NAS. The hepatic I/R injury model was generated by clamping the hepatic artery, portal vein, and common bile duct with a microvascular bulldog clamp for 30 min, and then removing the clamp and allowing reperfusion for 6 h. Morphologic changes and hepatocyte apoptosis were evaluated by hematoxylin-eosin (HE) and terminal deoxynucleotidyl transferase dUTP nick end labeling (TUNEL) staining, respectively. Activated caspase-3 expression was evaluated by immunohistochemistry and Western blot. The activation of aspartate aminotransferase (AST), malondialdehyde (MDA), and superoxide dismutase (SOD) was evaluated by enzyme-linked immunosorbent assay (ELISA). The data show that NAS rescued hepatocyte morphological damage and dysfunction, decreased the number of apoptotic hepatocytes, and reduced caspase-3 activation. Our work demonstrates that NAS ameliorates hepatic IR injury.

## 1. Introduction

Hepatic ischemia-reperfusion (I/R) injury, a major cause of liver damage during liver surgery and transplantation, triggers a series of molecular changes in hepatocytes, including insufficient ATP, increased production of reactive oxygen species (ROS), decline in ROS scavenging [1], and damage to mitochondria by accumulated free radicals, which perpetuates ROS generation. The overproduction of ROS leads to lipid peroxidation, mitochondrial membrane damage [2], release of cytochrome C (Cyt c) into the cytoplasm followed by caspase-3 activation, and finally, the triggering of hepatocyte apoptosis [3,4]. Therefore, identifying effective free-radical scavengers has great significance for the prevention and treatment of I/R injury. In addition, considering that *N-*acetylserotonin (NAS), but not serotonin or melatonin, promotes an early event of neurogenesis by activating BDNF tyrosine kinase (TrkB) receptors [5,6], together with fact that its brain distribution patterns are different from those of melatonin [7,8], we hypothesized that NAS may have functions other than serving as a precursor of melatonin.

Other researchers have shown that melatonin protects against liver injury related to ischemia/reperfusion [9,10], alcohol [11], and malarial infection [12]. However, the effect of NAS on hepatic I/R injury has not been reported. Additional studies have demonstrated that NAS acts as a temperature modulator [13], immune modulator [14], anti-depressant [15], analgesic [16], and inhibits pathological opening of mitochondrial permeability transition pores [17]. Recent research has shown that NAS also exhibits protective effects against peroxidative damage of neurons [18–20], lung epithelial cells [21], erythrocytes [22], testicular cells [23], retinal cells [24,25], and lymphocytes [26]. However, little is known about the effect of NAS on hepatic I/R injury. In the current study, we identified protective effects of NAS against hepatic I/R injury in animal models. We aim to develop new drug treatments for hepatic I/R injury, improve the efficacy of the treatment of hepatic ischemia-reperfusion injury, and offer experimental support for these innovations.

## 2. Results and Discussion

### 2.1. The Effect of NAS on Hepatocyte Morphology and Apoptosis

**Figure 1:**
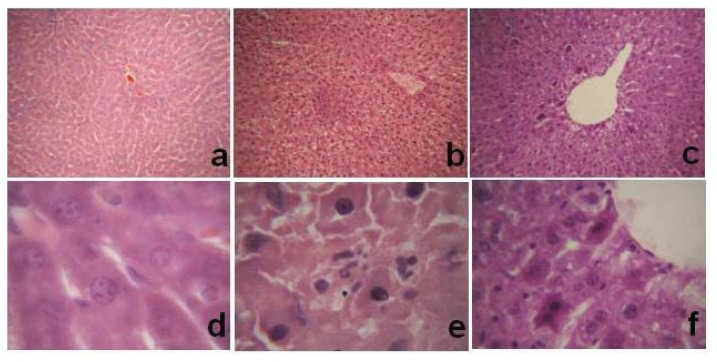
Effects of I/R and NAS treatment on hepatocyte morphology. (**a** and **d**): sham, the mice were subjected to the same surgical procedures except for liver I/R; (**b** and **e**): I/R, the mice underwent the liver I/R operation; and (**c** and **f**): I/R + NAS, NAS (5 mg/kg) was administered by i.p. injection 30 min before ischemia. Note the hepatocytes exhibiting ballooning hydropic degeneration and massive neutrophil infiltration in the I/R group. [(**a**–**c)**: 100×; (**d**–**f**): 1000×].

**Figure 2:**
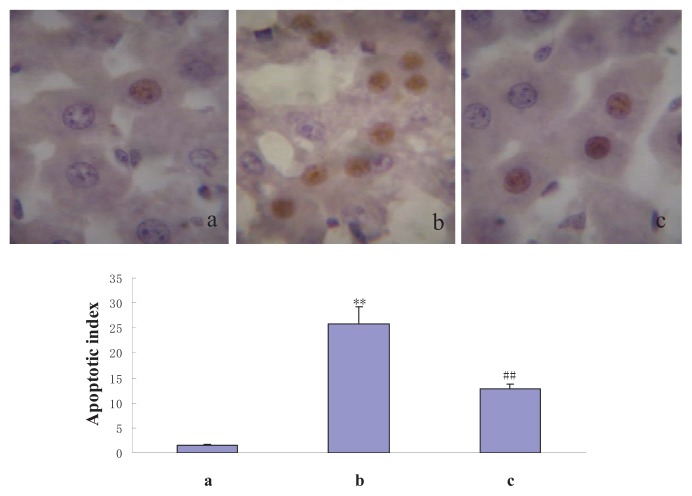
Effects of I/R and NAS treatment on apoptotic index. (**a**): sham, the mice subjected to all surgical procedures except for liver I/R; (**b**): I/R, the mice underwent the liver I/R operation; and (**c**): I/R + NAS, NAS (5 mg/kg) was administered by i.p. injection 30 min before ischemia. Note the significantly higher rate of apoptosis in the I/R group compared with the sham group (*p* < 0.01) and the lower rate in the I/R + NAS group compared with the I/R group (*p* < 0.01). Error bars represent SD (*n* = 6). The annotation ** indicates a *p* value < 0.01 *vs.* sham group. The annotation ^##^ indicates a *p* value < 0.01 *vs.* I/R group. [(**a**–**c**): 1000×].

To determine whether NAS protects a variety of hepatocytes from I/R injury, we constructed a liver I/R injury model by clamping the hepatic artery, portal vein, and common bile duct with a microvascular bulldog clamp for 30 min, and then removing the clamp and allowing reperfusion for 6 h. Frozen liver samples were cut into 10-μm-thick sections. Morphologic changes and apoptosis of hepatocytes were evaluated by hematoxylin-eosin (HE) and terminal deoxynucleotidyl transferase dUTP nick end labeling (TUNEL) staining, respectively. In the sham group, the hepatic lobules and cords were neatly arranged, and the hepatocyte nuclei were oval with uniform chromatin. There were occasional TUNEL-positive hepatocytes. In the I/R group, liver structure was disordered, the central and hepatic sinusoidal veins were congested, and hepatocytes exhibited marked ballooning hydropic degeneration, many acidophilic bodies, and massive neutrophil infiltration. Compared to the control group, there were many more TUNEL-positive hepatocytes around the portal area in the I/R group. In the I/R + NAS group, liver tissue damage was significantly reduced, the basic hepatic lobule structure was normal, some hepatocytes showed evidence of granular degeneration near the central vein, and there was no liver necrosis. The *AI* values of the three groups were 1.5 ± 0.3%, 25.8 ± 3.5%, and 12.7 ± 1.0% in the sham, I/R, and NAS groups, respectively. The *AI* of the I/R group was higher than that in either the sham or I/R + NAS groups (*p* < 0.01) (Figures 1 and 2).

### 2.2. The Effect of NAS on Serum Aspartate Aminotransferase (AST) Levels

**Figure 3:**
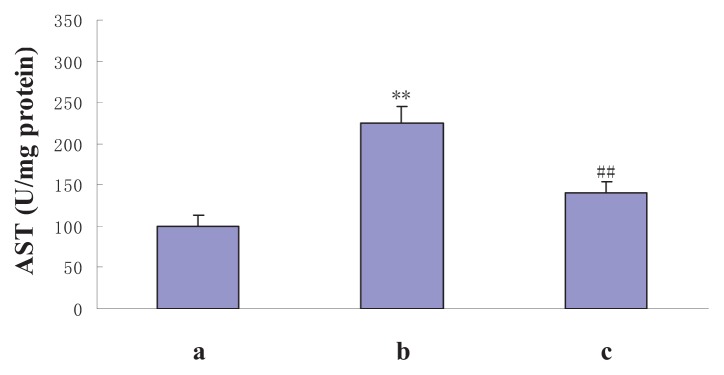
Effects of I/R and its treatment with NAS on AST. (**a**): sham, the mice were subjected to all surgical procedures except for liver I/R; (**b**): I/R, the mice underwent the liver I/R operation; (**c**): I/R + NAS, NAS (5 mg/kg) was administered by i.p. injection 30 min before ischemia. Note the significant increase in AST in the I/R group compared with the sham group (*p* < 0.01) and the decrease in the I/R + NAS group compared with the I/R group (*p* < 0.01). Error bars represent SD (*n* = 6). The annotation ** indicates a *p* value < 0.01 *vs.* sham group. The annotation ^##^ indicates a *p* value < 0.01 *vs.* I/R group.

Aspartate aminotransferase (AST) is mainly synthesized in the liver. Liver diseases such as ischemic injury and toxic injury may result in the leakage of AST into the circulation. Therefore the level of serum AST could be used as a biochemical marker to evaluate the role of NAS in rescuing hepatocyte cytolysis induced by liver I/R injury [27]. We found that with longer reperfusion times, the AST levels of the I/R and I/R + NAS groups increased, peaked at 6 h, and gradually decreased. Compared to the I/R group, AST levels were significantly lower in I/R + NAS groups (*p* < 0.01) (Figure 3).

### 2.3. The Effect of NAS on Oxidative Stress Following I/R Injury

Another critical factor in I/R injury is apoptosis mediated by superoxide dismutase (SOD). SOD, a critical enzyme in cellular protection, may act as a first line of defense against ROS, and malondialdehyde (MDA) was found to be a good indicator of the rate of lipid peroxidation [28,29].

**Figure 4:**
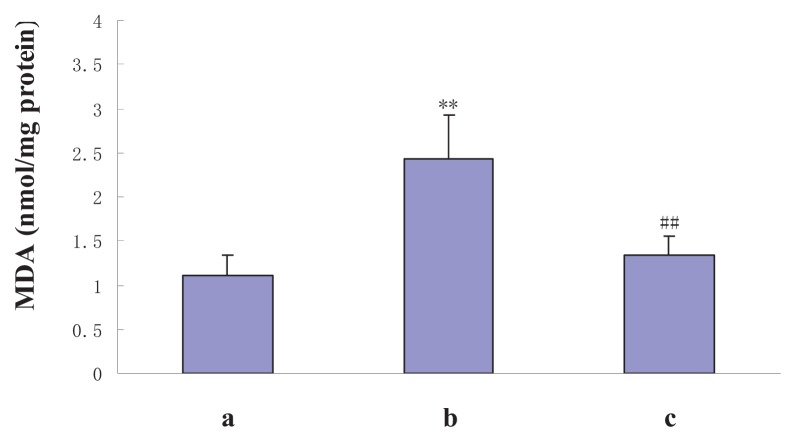
Effects of I/R and NAS treatment on MDA levels. (**a**): sham, mice were subjected to all surgical procedures except for liver I/R; (**b**): I/R, the mice underwent the liver I/R operation; and (**c**): I/R + NAS, NAS (5 mg/kg) was administered by i.p. injection 30 min before ischemia. Note the significant increase in MDA in the I/R group compared with the sham group (*p* < 0.01) and the decrease in the I/R + NAS group compared with the I/R group (*p* < 0.01). Error bars represent SD (*n* = 6). The annotation ** indicates a *p* value < 0.01 *vs.* sham group. The annotation ^##^ indicates a *p* value < 0.01 *vs.* I/R group.

**Figure 5:**
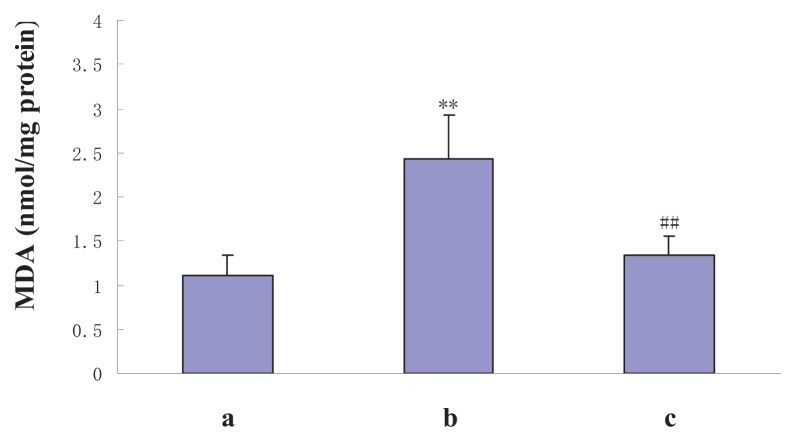
Effects of I/R and NAS treatment on SOD levels. (**a**): sham, mice were subjected to all surgical procedures except for liver I/R; (**b**): I/R, the mice underwent the liver I/R operation; (**c**): I/R + NAS, NAS (5 mg/kg) was administered by i.p. injection 30 min before ischemia. Note the significant decrease in SOD in the I/R group compared with the sham group (*p* < 0.01) and the increase in the I/R + NAS group compared with the I/R group (*p* < 0.01). Error bars represent SD (*n* = 6). The annotation ** indicates a *p* value < 0.01 *vs.* sham group. The annotation ^#^ indicates a *p* value < 0.05 *vs.* I/R group.

To determine whether NAS protects hepatocytes against oxidative stress damage, SOD and MDA concentrations in the liver homogenates of the sham and experimental groups were measured. SOD activities of the sham, I/R, and I/R + NAS groups were 104.6 ± 12.4, 68.6 ± 15.9 and 91.6 ± 13.9 U/mg protein, respectively. MDA contents were 1.1 ± 0.2, 2.4 ± 0.5 and 1.4 ± 0.2 nmol/mg protein, respectively. NAS administration caused a significant decrease in MDA content and increased SOD enzyme activity compared with the I/R group (*p* < 0.01) (Figures 4 and 5).

### 2.4. The Effect of NAS on Caspase-3 Activity and Liver Protein Expression

**Figure 6:**
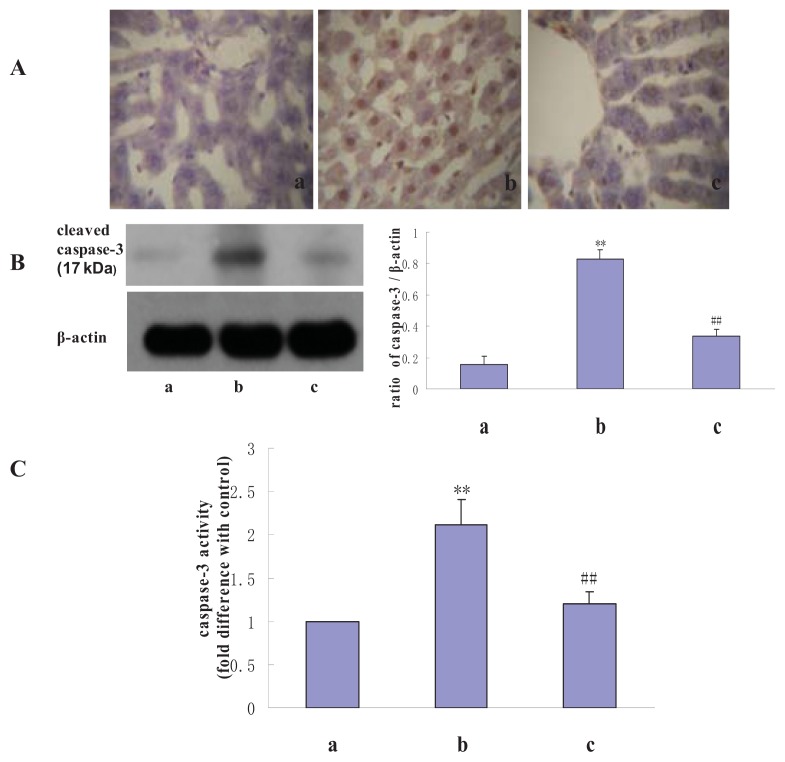
Effects of I/R and NAS treatment on caspase-3. (**A**): Cleaved caspase-3 expression as assessed by immunohistochemistry [(**a**–**c**): 400×]; (**B**): Cleaved caspase-3 expression as assessed by Western blot; and (**C**): Caspase-3 activity. (**a**): sham, mice were subjected to all surgical procedures except for liver I/R; (**b**): I/R, mice underwent the liver I/R operation; and (**c**): I/R + NAS, NAS (5 mg/kg) was administered by i.p. injection 30 min before ischemia. Note the significantly greater caspase-3 activity in the I/R group compared with the sham group (*p* < 0.01) and the lower activity in the I/R + NAS group compared with the I/R group (*p* < 0.01). The annotation ** indicates a *p* value < 0.01 *vs.* sham group. The annotation ^##^ indicates a *p* value < 0.01 *vs.* I/R group.

To further evaluate the mechanisms of I/R-induced hepatocyte injury, caspase-3 activity and protein expression in liver tissues were assessed by enzyme cleavage assay and Western blotting. Compared with the sham group, expression of cleaved caspase-3 in the liver tissues was higher in the I/R group. Treatment with NAS reduced cleaved caspase-3 levels compared with those in the I/R group. Results were similar for caspase-3 activity, *i.e.*, compared with controls, caspase-3 activity increased significantly in the I/R group. NAS significantly ameliorated the I/R-induced increase in caspase-3 activity, suggesting that NAS exerted an anti-apoptotic effect after I/R injury (*n* = 6, *p* < 0.01) (Figure 6).

### 2.5. Discussion

I/R injury refers to tissue damage caused when blood supply is restored after a period of ischemia. Hepatic I/R injury occurs in a variety of clinical conditions, including liver transplantation, circulating shock, disseminated intravascular coagulation, and liver surgery. I/R injury is mediated by a complex chain of events that involves ATP depletion, disruption of membrane integrity, ionic homeostasis alteration, ROS production, and cell death [30,31]. In this context, mitochondria may be important, given their critical roles in energy production, ROS generation, and apoptosis initiation during I/R [32]. Accumulating evidence indicates that ROS are important mediators in hepatic I/R injury, and several different antioxidants and inhibitors of ROS generation have been shown to attenuate hepatocyte ROS-induced cell death during hypoxia and I/R injury [33,34].

Melatonin is known to have antioxidant properties. It has previously been demonstrated to attenuate I/R injury in several organs, including liver, brain, myocardium, kidney, pancreas, and retina [35–39].

NAS is a precursor of melatonin that is mainly formed from 5-hydroxytryptamine via acetylation in the pineal gland cells, and it is subsequently methylated by hydroxyindole-*O*-methyltransferase to synthesize melatonin. NAS, a melatonin precursor, is a potent scavenger of ROS, a strong antioxidant, and an inhibitor of cytochrome c release from mitochondria. The antioxidant effect of NAS is independent from that of melatonin and is 5 to 20 times stronger [25,40], while the effective antioxidant dose of melatonin to protect the liver from oxidative stress during malaria is 20 times lower than that of two known antioxidants, vitamin C and vitamin E. Thus, we hypothesized that NAS is a stronger antioxidant than NAS, melatonin, vitamin C, or vitamin E [12]. The antioxidant effects of NAS might underlie its cognition-enhancing [41], analgesic [16], and anti-depressant effects [15]. NAS and its derivatives might be useful in protecting against oxidative stress-related disorders, including cell death, mutagenesis, aging, and diseases such as sepsis, cancer, post-ischemic trauma, Alzheimer’s disease, and parkinsonism. Oxenkrug and colleagues demonstrated for the first time the anti-aging effect of NAS [42]. Using the membrane peroxidative damage model of brain, liver, and kidney homogenates by 400 μg/mL bacterial lipopolysaccharide (LPS), Stuss [43] reported that both melatonin and NAS decreased LPS-stimulated LPO. Furthermore, other researchers showed that the protective effect of NAS was greater than that of melatonin in erythrocytes [22], testicular cells [23], retinal cells [24,25], and lymphocytes [26].

Taken together, evidence indicates that NAS has antioxidant properties *in vitro*. Whether NAS has protective effects against visceral organ I/R injury has not been reported. However, NAS protects against iron-, copper-, H_2_O_2_-, and LPS-induced lipid peroxidation better than melatonin, and melatonin has been demonstrated to attenuate I/R injury in many organs including liver, brain, myocardium, kidney, pancreas, and retina. Therefore, we hypothesized that NAS has a protective effect against hepatic I/R injury.

First, we assessed the effects of NAS on hepatocyte morphology and function in a mouse model of I/R injury. We found that NAS normalized hepatocyte appearance and reduced serum levels of AST, both enzymatic markers of hepatic damage.

Increasing evidence shows that ROS release is one of the earliest and most important features of tissue injury after ischemic organ reperfusion, and is a major contributor to hepatocyte death [44]. Under normal circumstances, ROS are counteracted by antioxidant enzymes, including SOD and glutathione peroxidase and reductase (GSH-Px and GSH-Rd). However, the body’s antioxidant enzyme system is insufficient to handle rapid-onset conditions such as I/R injury, during which high levels of ROS are produced [45]. SOD is a critical cellular protection enzyme that is a first line of defense against ROS, and MDA is a good indicator of the lipid peroxidation rate. In the present study, MDA levels were significantly increased by I/R, and NAS significantly inhibited MDA production. Conversely, SOD levels were decreased by I/R, and this was reversed by NAS. Based on these findings, we suggest that NAS could reverse the harmful imbalance between oxidants and antioxidants.

To verify the mechanism underlying NAS’s protective effect on hepatic I/R injury, we also investigated whether NAS played a role in inhibiting apoptosis. Though I/R injury increased caspase-3 activity and the number of TUNEL-positive hepatocytes, NAS treatment reversed these changes.

## 3. Experimental Section

### 3.1. Chemicals

NAS was obtained from Sigma (St. Louis, MO, USA). Anti-cleaved caspase-3 and anti-β-actin antibodies were purchased from Cell Signaling Technology, Inc. (Beverly, MA, USA) and Santa Cruz Biotechnology (Santa Cruz, CA, USA), respectively. Biotinylated anti-rabbit or -mouse immunoglobulin G (IgG), peroxidase-conjugated streptavidin, and horseradish peroxidase-conjugated goat anti-rabbit and -mouse IgG were purchased from Boster Biotechnology (Wuhan, China). The caspase-3 activity assay kit was purchased from Beyotime Institute of Biotechnology (Haimen, China). The enhanced chemiluminescence (ECL) system was purchased from Amersham Pharmacia Biotech (Piscataway, NJ, USA). The TUNEL apoptosis and DAB (3,3-diaminobenzidine) kits were from Boster Biotechnology. AST, MDA, and SOD kits were obtained from NanJing JianCheng Bioengineering Institute (Nanjing, China). OCT (optimum cutting temperature) compound was purchased from Tissue Tek (Sakura Finetek, Torrance, CA, USA).

### 3.2. Animals

Eighteen adult male Kunming mice weighing 25–30 g were used. The animals were housed in the Weifang Medical University animal facility under specific pathogen-free conditions and received humane care according to the University guidelines. Under 1% sodium pentobarbital (40 mg/kg) anesthesia by intraperitoneal (i.p.) injection, ischemia was induced by clamping the hepatic vessels and bile duct using a noninvasive vascular clip. The success of the model was apparent once the liver color turned from red to dark purple. After 30 min of ischemia, the clip was removed to allow hepatic reperfusion. Body temperature was kept constant with a heating pad during surgical procedures.

In preliminary experiments, we noted hepatic cord deformation, hepatocyte necrosis, and massive neutrophil infiltration at 6 h postreperfusion. AST levels reached a peak at 6 h, illustrating that liver tissue damage was most severe at that time. Therefore, mice were sacrificed 6 h after reperfusion.

### 3.3. Experimental Design

Mice were randomly assigned to one of three groups: sham operation (sham), I/R, and I/R + NAS (*n* = 6 per group). In the preliminary experiment, we found that 20 mg/kg NAS had less protective effect than 10 or 5 mg/kg NAS. Although the protective effects of 10 and 5 mg/kg were similar, we chose to use 5 mg/kg NAS in this experiment based on the principle of minimal use of clinical drugs. Sham group mice were subjected to the surgical procedures, but the clamp was not placed, and they were kept under anesthesia for an equivalent duration. NAS (5 mg/kg) or saline was administered i.p. 30 min before ischemia in the I/R + NAS and I/R groups. The rationale for the particular pre-treatment experimental design is based on our previous studies of the protective effect of drugs/agents in ischemic brain injury and other diseases [46–48].

### 3.4. Specimen Collection

Mice were euthanized by sodium pentobarbital 6 h after reperfusion. Blood samples were collected immediately from the heart. After centrifugation of whole blood (4000× *g*, 4 °C for 5 min), serum was extracted and stored at −70 °C until analysis. Liver tissues were removed and rinsed in cold 0.1 M phosphate-buffered saline (PBS, pH 7.4). Portions of the liver samples were fixed in 10% formalin for HE, immunohistochemistry, or TUNEL staining. The remaining samples were snap-frozen in liquid nitrogen and stored at −70 °C for SOD and MDA measurements.

### 3.5. Histopathological Examination

Livers from mice in all groups were rapidly excised and fixed in 10% formalin for at least 24 h, then transferred to 30% sucrose in 0.1 M PBS at 4 °C for 1 day. Then they were mounted in OCT compound and frozen, and serial 10-μm-thick sections were cut on a cryostat, mounted on microscope slides, and stored at −20 °C. For histological analysis, the sections were examined by HE staining in a standard manner, and morphological changes in hepatocytes were assessed under light microscopy.

### 3.6. Immunohistochemistry Staining

The indirect immunoperoxidase method was used to localize cleaved caspase-3 in frozen sections. We employed a Polymer Detection System for immunohistological staining, and the DAB kit was used according to the manufacturer’s guidelines as described previously [17,18]. Endogenous peroxidase was inactivated by immersing the sections in hydrogen peroxide for 10 min, rinsing in PBS, then blocking with 1% goat serum for 30 min at room temperature. The sections were incubated overnight with polyclonal antibodies against cleaved caspase-3 for 45 min at 37 °C, followed by peroxidase-conjugated streptavidin for 45 min at 37 °C. All sections were counterstained with hematoxylin.

### 3.7. Terminal dUTP Nick End Labeling Assay (TUNEL)

The TUNEL assay is a method of demonstrating apoptotic cell death. To evaluate the extent of hepatocyte apoptosis, TUNEL staining was performed in frozen sections. Briefly, following postfixing in 10% formalin for 15 min, the sections were digested with 20 μg/mL proteinase K in PBS for 15 min at room temperature, and endogenous peroxidases were inactivated by immersing the sections in hydrogen peroxide for 10 min before they were rinsed in PBS. Then the sections were incubated with TUNEL reaction mixture for 2 h at 37 °C, rinsed three times with PBS, and blocked with blocking solution for 30 min at room temperature. The sections were further incubated with biotinylated anti-digoxigenin conjugate and avidin-biotin complex (ABC) for 30 min at 37 °C, respectively. DAB was used as a chromogen, and sections were counterstained with hematoxylin. For control sections, PBS was used instead of the TUNEL reaction mixture.

Apoptotic hepatocytes were counted in six random high-power fields (×40 objective). The degree of apoptosis was assessed using the apoptosis index (*AI*), which is calculated by the following formula: AI=(the number of apoptotic hepatocytes/total number of hepatocytes)×100%.

### 3.8. Western Blotting Analysis

Tissues were lysed in RIPA buffer, cytoplasmic proteins were extracted, and protein concentration was determined by Lowry’s method. Next, proteins (50 μg/sample) in sodium dodecyl sulfate (SDS)-loading buffer were boiled for 5 min, separated by 10% SDS-polyacrylamide gel electrophoresis, and transferred to PVDF membranes. The membranes were blocked with 5% nonfat milk in Tris-buffered saline (TBS) and subsequently incubated with cleaved caspase-3 (1:500, cell signaling) or β-actin primary antibody, followed by horseradish peroxidase-conjugated goat, anti-rabbit or -mouse IgG secondary antibody, and bands were visualized with an enhanced chemiluminescence (ECL) system according to the manufacturer’s instructions. Densitometric analyses of the Western blot bands were performed using optical density scanning and ImageJ software (NIH, Bethesda, MD, USA). The relative ratio of target protein optical density values to those of the internal reference protein β-actin were determined for each group (mean ± standard deviation).

### 3.9. Serum AST Analysis

AST serum levels were detected by commercial kits according to the manufacturer’s instructions.

### 3.10. MDA and SOD Assays

The frozen samples were homogenized and centrifuged. The supernatants were collected for MDA and SOD analysis. The measurements were obtained spectrophotometrically with commercial kits according to the manufacturer’s recommendations. MDA levels are given in nmol/mg protein.

SOD activity was assayed using the nitroblue tetrazolium (NBT) method. NBT was reduced to blue formazan by superoxide, which has strong absorbance at 560 nm. One unit (U) of SOD is defined as the amount of protein that inhibits the NBT reduction rate by 50%. SOD activity was expressed as U/mg protein.

### 3.11. Caspase-3 Activity Assay

Liver samples were collected and rinsed with PBS, lysed in lysis buffer for 15 min on ice, then centrifuged at 20,000× *g* for 10 min at 4 °C. Caspase-3 activity in the supernatant was assayed with commercially available kits obtained from the Beyotime Institute of Biotechnology according to the manufacturer’s instructions. All experiments were carried out in triplicate.

### 3.12. Statistical Analysis

All data were expressed as mean ± SD (standard deviation) and were analyzed using SPSS 11.0 software (SPSS, Chicago, IL, USA). *p* < 0.05 was considered statistically significant.

## 4. Conclusions

The present study demonstrates that NAS reduced serum AST levels, reduced histological liver lesions, and attenuated caspase-3 activity, indicating that NAS ultimately inhibited hepatocyte apoptosis following I/R. Beneficial effects are correlated with inhibited ROS production and the maintenance of SOD activity. Thus, NAS ameliorated hepatic I/R injury by reversing the imbalance between oxidants and antioxidants and inhibiting hepatocyte apoptosis.
